# Including pork in the Mediterranean diet for an Australian population: Protocol for a randomised controlled trial assessing cardiovascular risk and cognitive function

**DOI:** 10.1186/s12937-017-0306-x

**Published:** 2017-12-22

**Authors:** Alexandra T. Wade, Courtney R. Davis, Kathryn A. Dyer, Jonathan M. Hodgson, Richard J. Woodman, Hannah A. D. Keage, Karen J. Murphy

**Affiliations:** 1 Alliance for Research in Exercise, Nutrition and Activity, School of Health Sciences, University of South Australia, GPO Box 2471, Adelaide, South Australia 5001 Australia; 2 School of Medicine and Pharmacology, Faculty of Medicine, Dentistry and Health Sciences, University of Western Australia, 35 Stirling Highway, Perth, WA 6000 Australia; 3 School of Medical and Health Sciences, Edith Cowan University, Perth, WA Australia; 4 Flinders Centre for Epidemiology and Biostatistics, Flinders University, GPO Box 2100, Adelaide, South Australia 5001 Australia; 5 Cognitive Ageing and Impairment Neurosciences, School of Psychology, Social Work and Social Policy, University of South Australian, GPO Box 2471, Adelaide, SA 5001 Australia

**Keywords:** Mediterranean diet, CVD, Cognitive function, Randomised controlled trial

## Abstract

**Background:**

The Mediterranean diet is characterised by the high consumption of extra virgin olive oil, fruits, vegetables, grains, legumes and nuts; moderate consumption of fish, poultry, eggs and dairy; and low consumption of red meat and sweets. Cross sectional, longitudinal and intervention studies indicate that a Mediterranean diet may be effective for the prevention of cardiovascular disease and dementia. However, previous research suggests that an Australian population may find red meat restrictions difficult, which could affect long term sustainability of the diet.

**Methods:**

This paper outlines the protocol for a randomised controlled trial that will assess the cardiovascular and cognitive benefits of a Mediterranean diet modified to include 2-3 weekly serves of fresh, lean pork. A 24-week cross-over design trial will compare a modified Mediterranean diet with a low-fat control diet in at-risk men and women. Participants will follow each of the two diets for 8 weeks, with an 8-week washout period separating interventions. Home measured systolic blood pressure will be the primary outcome measure. Secondary outcomes will include body mass index, body composition, fasting blood lipids, C-reactive protein, fasting plasma glucose, fasting serum insulin, erythrocyte fatty acids, cognitive function, psychological health and well-being, and dementia risk.

**Discussion:**

To our knowledge this research is the first to investigate whether an alternate source of protein can be included in the Mediterranean diet to increase sustainability and feasibility for a non-Mediterranean population. Findings will be significant for the prevention of cardiovascular disease and age-related decline, and may inform individuals, clinicians and public health policy.

**Trial registration:**

ACTRN12616001046493. Registered 5 August 2016.

## Background

As the population ages the prevalence and impact of chronic age-related diseases and disorders is predicted to increase dramatically [1]. In the elderly, dementia and cardiovascular disease (CVD) are amongst the greatest contributors to death and disability worldwide, and their costs are expected to double and triple respectively over the next 20 years [2–4].

Mediterranean populations in southern Europe, such as Greece and Italy, exhibit significantly lower rates of mortality from CVD and dementia than populations in northern Europe and the United States [5]. It has been suggested that this disparity is due to diverse dietary patterns across populations, and that a Mediterranean diet may be protective against CVD [6, 7]. As cardiovascular health is a significant predictor of dementia [8, 9], the Mediterranean dietary pattern may also be responsible for lower rates of dementia.

A traditional Mediterranean diet is characterised by a high intake of extra virgin olive oil (EVOO), vegetables, fruits, cereals, nuts, pulses and legumes; a moderate intake of fish, poultry, dairy and red wine; and a low intake of eggs, red and processed meat, and sweet and processed foods [10]. The Mediterranean dietary pattern is therefore rich in bioactive nutrients and phytochemicals such as monounsaturated fatty acids, polyunsaturated fatty acids (including omega-3 s), polyphenols and flavonoids, vitamins, minerals, antioxidants and fibre. Individually, these dietary components are associated with improved cardiovascular health [11–14]. However, single nutrients are not consumed in isolation, but collectively as a dietary pattern. As such, synergistic relationships between nutrients may enhance these benefits [15–17].

When compared with other dietary patterns a Mediterranean diet has consistently improved indicators of cardiovascular health. For example, a Mediterranean diet has been shown to improve blood pressure, insulin sensitivity, lipid profiles and markers of inflammation [18–20], while reducing risk of cardiovascular events by 30% [21].

While Mediterranean populations who follow a Mediterranean diet exhibit lower risk of CVD and dementia [15, 22–25], few studies have investigated whether the diet can be successfully adopted in populations beyond the Mediterranean Sea. Notably, the MedLey study examined the effects of a Mediterranean diet over 6 months and found that an older Australian population was capable of adopting the Mediterranean diet [26]. However, participants indicated that one of the most difficult aspects of following the diet was restricting red meat intake [27].

A traditional Mediterranean diet is typically low in red meat products and Mediterranean dietary guidelines recommend consuming less than two serves of red meat per week [28, 29]. A recent review has reported that a traditional Mediterranean diet includes 105 g, or 0.5 to 0.75 standard serves of meat and meat products per day [10]. This figure is inclusive of all processed and unprocessed red and white meat products. In contrast, Australians over the age of 19 consume an average of 184 g of meat per day, or 1.7 serves of lean meats and alternates, with red meat contributing the largest proportion of this (38%) [30]. Australians are amongst the highest consumers of meat in the world [31]. Therefore, it is not surprising that restricting red meat may prove difficult. Countries with comparatively high meat intakes, such as the United States and United Kingdom, may experience similar difficulties (OECD, 2016). As socio-cultural norms and palatability are key determinants of a dietary intervention’s sustainability [32], non-Mediterranean populations may then be more likely to adhere to a diet containing more red meat.

Prospective cohort studies have drawn attention to significant associations between red meat and poor health outcomes, including bowel cancer and CVD [33]. Proposed mechanisms point to the pro-oxidative properties of heme iron found in red meat. However, recent investigations highlight the mediating effects of other dietary components on oxidation [34]. For example, consuming EVOO with red meat has been shown to attenuate lipid oxidation and the production of free radicals [35]. When vitamin E, found in green leafy vegetables and nuts, is consumed in combination with EVOO and red meat, lipid oxidation is further inhibited [35]. Moreover, the European Prospective Investigation into Cancer and Nutrition (EPIC) study reported significant interactions between red meat and dietary fibre [36]. Specifically, individuals consuming red meat every day had a reduced risk of developing colorectal cancer if they also consumed at least 26 g of dietary fibre each day – that is, less dietary fibre than is provided by the Mediterranean diet [10]. The potentially harmful effects of red meat may then be mitigated when consumed within a Mediterranean dietary pattern.

Australia’s most frequently consumed red meat products are beef and pork [31]. In terms of nutritional value, fresh lean beef and pork have similar nutrient profiles. However, fresh lean pork contains considerably less heme iron than beef [37] and less saturated fat per 100 g. Pork may then be an appropriate addition to a Mediterranean diet, which is typically low in saturated fat and heme iron sources [10]. Our previous research indicates that substituting beef and chicken with pork may be effective for improving cardiometabolic health markers, including weight, body mass index (BMI), percentage body fat, fat mass and abdominal fat, without negatively affecting blood lipids, insulin or glucose [38]. Further, when compared with ruminant meats such as beef and some methods of aquaculture, pork production is associated with significantly less agricultural greenhouse gas emissions [39].

At present, there are no studies to our knowledge that have investigated the substitution of meat protein sources in the Mediterranean diet to increase variety and sustainability for the target population and environment. The current study therefore aims to evaluate the cardiovascular and cognitive benefits of a Mediterranean diet, supplemented with fresh, lean pork against a low-fat diet. A low-fat control diet has been chosen due to its continued recommendation for the clinical management and reduction of cardiovascular risk [40]. Further, including a low-fat control will enable comparisons of our findings against larger studies of the same design, such as the Prevención con Dieta Mediterránea (PREDIMED) trial. Due to the pressing need to reduce the impact of CVD and dementia, the modified Mediterranean diet will be evaluated for its potential to improve cardiovascular health and cognitive risk in an at-risk population.

## Methods

### Recruitment

Details of the current study protocol are provided in Table 1. Volunteers aged between 45 and 80 who are at risk of developing CVD will be recruited via electronic and paper advertisements.

**Table 1:** World Health Organisation Trial Registration Data Set

Data category	Information
Primary registry and trial identifying number	Australian New Zealand Clinical Trials Registry (ANZCTR) ACTRN12616001046493
Date of registration in primary registry	5 August, 2016
Secondary identifying numbers	University of South Australia Human Ethics Committee 35,562. Pork CRC 3B-113.
Source(s) of monetary or material support	Australian Pork Cooperative Research Council
Primary sponsor	Dr Karen J Murphy
Secondary sponsors	Alexandra T Wade, Courtney R Davis, Kathryn A Dyer, Jonathan M Hodgson, Richard J Woodman, Hannah A.D. Keage
Contact for public queries	Dr Karen J Murphy Phone: +61 8202 2097. Email: Karen.Murphy@unisa.edu.au Postal address: University of South Australia, GPO Box 2471, Adelaide SA 5001
Contact for scientific queries	Dr Karen J Murphy, Senior Research Fellow, Dietitian, Alliance for Research in Exercise, Nutrition and Activity, University of South Australia. Phone: +61 8202 1033. Email: Karen.Murphy@unisa.edu.au Postal address: University of South Australia, GPO Box 2471, Adelaide SA 5001
Public title	Effect of a Mediterranean diet with fresh lean Australian pork on blood pressure, cardiovascular risk factors and cognition, mood and psychological wellbeing in high risk individuals
Scientific title	Including pork in the Mediterranean diet for an Australian population: Protocol for a randomised controlled trial assessing cardiovascular and cognitive outcomes
Countries of recruitment	Australia
Health condition(s) or problem(s) studied	Risk of cardiovascular disease (CVD)
Intervention(s)	Active comparator: Mediterranean diet including fresh, lean pork Control comparator: Low-fat diet
Key inclusion and exclusion criteria	Inclusion criteria: adult (45-80 years) with elevated blood pressure (>120 mmHg) with at least two other risk factors for CVD. Exclusion criteria: antihypertensive medication, dietary intolerances that may restrict consumption of intervention diets, current CVD, type 2 diabetes mellitus, malignancy, liver, kidney, gastrointestinal or respiratory disease, current diagnosis of dementia or use of antidepressants, anxiety or neurological medications
Study type	Type: Interventional Allocation: Randomised block allocation, parallel cross-over design Primary purpose: prevention
Date of first enrolment	15 February 2017
Target sample size	31
Recruitment status	Complete
Primary outcome(s)	Home measured systolic blood pressure
Key secondary outcomes	Body mass index (BMI), body composition, fasting blood lipids, C-reactive protein, fasting plasma glucose, fasting serum insulin, erythrocyte fatty acids, cognitive function, psychological health and well-being, and dementia risk.

To meet eligibility criteria, volunteers must have elevated systolic blood pressure above 120 mmHg and at least two other risk factors for CVD, including: a BMI ≥ 25 kg/m2; elevated fasting total cholesterol (≥5.5 mM), triglycerides (≥2.0 mM), low-density lipoprotein (LDL) (≥3.5), or low levels of high-density lipoprotein (HDL) (≤0.9 for men and ≤1.0 for women); impaired fasting glucose tolerance (between 6.1 and 7.8 mmol/L); and/or a family history (up to one generation) of CVD or type 2 diabetes mellitus (T2DM). Exclusion criteria include: antihypertensive medication; smoking; current CVD or angina; current or recent (within 6 months) malignancies; respiratory disease; gastrointestinal disease; kidney disease; T2DM; a current or previous traumatic head or brain injury; a current neurological or psychiatric condition; antidepressant or anxiety medication; a current diagnosis of Alzheimer’s disease or dementia; or supplemental omega-3 > 1000 mg daily.

Eligibility will be assessed through a diet and lifestyle questionnaire (DLQ) and screening visit at the Sansom Institute for Health Research Clinical Trial Facility (SIHR CTF), Adelaide, South Australia. The DLQ includes medical history, medications and supplements, family medical history, and dietary aversions, allergies and intolerances. Volunteers deemed eligible on the basis of their DLQ will be screened to measure systolic blood pressure, diastolic blood pressure, heart rate, height and weight, fasting cholesterol and fasting glucose. The Addenbrooke’s Cognitive Exam-Revised (ACE-R) will also be administered to detect pre-existing dementia and mild cognitive impairment (MCI).

### Design

**Fig. 1:**
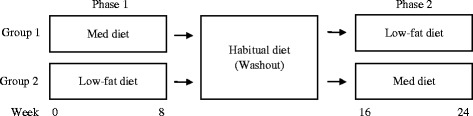
Title: 24-week cross-over design trial

A 24-week cross-over design trial will compare a Mediterranean diet intervention with a low-fat diet intervention. Each participant will complete both dietary interventions for 8 weeks and the two intervention phases will be separated by an 8-week washout period (See Fig. 1). An independent staff member of the SIHR will perform the block randomisation procedure. Participants will be allocated to one of two groups using, stratified by age and gender, to determine the order in which they undertake the interventions. Allocation concealment will ensure that study personnel and volunteers are not aware of the starting intervention prior to enrolment. During the washout period participants will return to their habitual diet to prevent cross-contamination of effects between dietary phases.

By reducing potential between-subject differences a cross-over design reduces the effects of potential confounding variables, and therefore enhances internal validity of the design. Further, performing repeated measures on the same individual allows for an estimation of an individual’s true value, measurement error and treatment effect, meaning that fewer participants will be required to detect a treatment effect. An 8-week intervention period has been chosen as randomised controlled trials (RCTs) of similar length have found that nutrients contained within the Mediterranean diet can improve both cardiovascular and cognitive function [41–43]. Eight weeks is expected to be an adequate washout period [44]. However, potential carryover effects will be assessed by testing for intervention*phase interactions.

### Dietary interventions

Neither diet will restrict energy intake. Instead, participants will be advised to self-regulate their food intake and consume foods ad-libitum.

#### Low-fat diet

Guidelines for the low-fat diet are based on the PREDIMED study, which compared two variations of a Mediterranean diet with a low-fat control diet [45]. Participants will be advised to make adjustments to their habitual diet in order to reduce total fat intake. Specifically, high fat foods, including all types of oil, butter, margarine, processed and high fat meats, nuts, chocolates, cakes, pastry, and high or full fat dairy, will be replaced with low-fat alternatives, such as breads and cereals, legumes, rice, fruits and vegetables and low-fat varieties (e.g. low-fat dairy, low-fat sauces). Daily limits will be set for oil (no more than 20 ml), butter and margarine (no more than two teaspoons), and participants will be instructed to remove visible fat and skin from meat and fish before cooking.

#### Mediterranean diet

Guidelines for the Mediterranean diet are adapted from Estruch et al. (2013) for an Australian food supply:

- Minimum of one tablespoon (20 mL) of EVOO per day;
- ≥2–3 daily servings of fresh fruit (one serve = 150 g fresh, 30 g dried, or one cup canned in natural juice);
- ≥3 weekly servings of legumes (one serve = 75 g);
- 2-3 weekly servings of fresh, lean pork (one serve = 100 g cooked)
- ≥3 weekly servings of fish and seafood (at least one serving of oily fish) (one serve = 100 g cooked);
- ≥5 weekly serving of raw or roasted nuts or seeds, without added salt, sugar or chocolate (one serve = 30 g; 7.5 g hazelnuts, 15 g walnuts, 7.5 g almonds supplied for each serve);
- Ad-libitum consumption of wholegrain cereal products (bread, pasta, rice, cereal) and dairy
- Ad-libitum consumption of eggs, not to exceed 6 serves per week (1 serve = approx. 70 g or 1 egg)
- Select white meats (poultry without skin) instead of red meats or processed meats;
- Limit consumption of red and cured meat (remove all visible fat) to ≤1 serve/week (one serve of red meat/cured ham = 100 g);
- Limit consumption of chocolate to ≤1 serve/week (one serve of chocolate = 50 g);
- Use EVOO for cooking and dressing vegetables and salad;
- Cook regularly (at least twice a week) with a tomato based sauce (EVOO, tomato, garlic and onion);
- Dress vegetables, pasta, rice and other dishes with EVOO, tomato, garlic and onion sauce;
- Eliminate or limit the consumption of cream, butter, margarine, cold meat, pate, duck, carbonated and or sugared beverages, pastries, commercial bakery products (cakes, donuts, cookies), desserts (puddings), French fries, potato crisps, sweets;
- For usual drinkers, red wine is recommended as the main source of alcohol with a maximum of two standard drinks per day (200 mL = two standard drinks) [46]

Participants will be advised to consume pork in place of chicken and red meat ensure that total meat consumption does not exceed 400 g per week.

To assist with adherence, the following foods will be provided each week: 375 ml EVOO; 250 g of fresh, lean pork; 150 g raw, unsalted almonds, walnuts and hazelnuts; 225 g (net weight) of canned chickpeas, red kidney beans, 4-bean mix and lentils; 95 g of canned tuna and 95 g of canned salmon.

#### Dietetic counselling

To increase adherence and retention participants will have regular contact with a dietitian. At the beginning of each phase, participants will meet with the dietitian to discuss dietary guidelines in detail. For the Mediterranean diet, participants will be provided with a set of resources, including Mediterranean dietary guidelines (Appendix 1), recommendations on how to include pork in the diet (Appendix 2), education on serving sizes and a Mediterranean diet recipe book. For the continual assessment of adherence, and to encourage familiarity of foods and serving sizes associated with a Mediterranean diet, participants will also be given a semi-quantitative checklist (Appendix 3) to be filled in daily, using a tick system (1 tick = 1 serve), and returned at bi-weekly dietetic visits. While on the low-fat diet, similar dietetic resources will be provided, including a set of low-fat diet guidelines (Appendix 4), education on fat content and a food label reading guide (Appendix 5) to consider when selecting packaged foods.

Throughout each intervention phase participants will attend bi-weekly dietetic visits to discuss their progress, food intake, challenges and any adverse effects. These visits will also include weight measurement to determine body mass, weight loss or weight gain. In the case of weight loss or gain the dietitian will ensure that participants are eating until satiated and discuss mindful eating and portion sizes. If a participant is having trouble adhering to the guidelines the dietitian will discuss strategies and work with the participant to set SMART goals (S, specific; M, measureable; A, achievable; R, realistic; T, time based) for the next 2 weeks.

### Outcome measures

#### Home blood pressure

Home measured systolic blood pressure will be the primary outcome measure. For the reliable assessment of hypertension, home measured blood pressure is comparable to ambulatory blood pressure, and has stronger predictive power than clinic blood pressure [47–49].

Participants will receive training on self-administered blood pressure measurement and will be instructed to measure their systolic and diastolic blood pressure and heart rate every morning, afternoon and evening for 6 days at the four assessment points over the course of the trial. Participants will be provided with a clinically validated A&D Company Ltd. digital blood pressure monitor (model UA-767). Measurements will be taken at a consistent time each day after a five-minute rested period in the seated position. Three consecutive readings are to be taken spaced at least 1 min apart, as per Bondonno et al. (2015) and participants will be advised to avoid caffeine and alcohol for 1 h prior to measurement, and food and exercise for 30 min prior.

#### Secondary outcome measures and covariates

Over the four assessment time points, home measured diastolic blood pressure and heart rate, clinic blood pressure and heart rate, BMI, waist-to-hip ratio, body composition, fasting blood lipids, C-reactive protein (CRP), fasting plasma glucose, fasting serum insulin, erythrocyte fatty acids, cognitive function, psychological health and well-being, and dementia risk will be examined as secondary outcomes.

Clinic blood pressure will be measured using an Omron Healthcare Co. digital blood pressure monitor (model 1A1B Hem-7000-CIL). The same protocol as for home blood pressure measurement will be followed, where participants will be seated for 5 min and three measurements will be taken spaced at least 1 min apart. Waist and hip circumference will be measured to determine waist-to-hip ratio. Body composition, including percentage body fat, lean mass and abdominal adiposity will be measured using dual-energy x-ray absorptiometry (DEXA). Fasting venous blood will be collected through venepuncture and analysed at an external NATA accredited laboratory using standard procedures. Erythrocyte fatty acids will be measured using direct transesterification as described by Tu et al. (2013) once further funding has been secured. Whole blood will be centrifuged (4 °C, 4000 rpm, 10 min) to separate erythrocytes from spare plasma, both of which will be frozen at −20 °C and then stored at −80 °C. APOEε4, an indicator of increased risk for AD, will be determined using the TaqMan® SNP Genotyping assay kit (Applied Biosystems, Warrington, UK) [50]. For explorative purposes, a faecal sample will also be collected to examine the effects of a Mediterranean diet on gut microbiota. Faecal samples will be frozen at −20 °C and then stored at −80 °C until further funding has been secured.

Cognitive function will be assessed across memory, processing speed and executive function using a selection of tests from the Cambridge Automated Neuropsychological Test Automated Battery (CANTAB). These particular cognitive domains will be examined due to their vulnerability to aging and cardiovascular health, and their demonstrated sensitivity to short term nutritional interventions [51–56]. The battery of tests will include Motor Orientation Task (MOT), Paired Associates Learning (PAL), Delayed Matching to Sample (DMS), Verbal Recognition Memory (VRM), Reaction Time (RTI), Rapid Visual Information Processing (RVIP), Spatial Working Memory (SWM), One Touch Stockings of Cambridge (OTS) and Attention Switching Task (AST).

A computerised mode of testing has been chosen to increase accuracy and reliability, especially where sensitivity to processing speed is critical [57]. To limit the effect of learning over repeated measures, the CANTAB offers parallel versions of each test. For the chosen tests, test re-test reliability (r) ranges from *r* = 0.54 (RTI) to *r* = 0.87 (PAL) [58]. To examine potential measurement error, especially where the test-retest coefficient is less than *r* = 0.7, learning effects can be determined using standardised scores and standard errors provided by Cambridge Cognition [58].

The Karolinska Sleepiness Scale (KSS) will be administered prior to cognitive testing to gain insight into sleep quality and sleepiness, which may impair cognitive function. The KSS is a 9-point scale that asks participants to rate their current level of sleepiness where 1 = Extremely alert, 2 = Very alert, 3 = Alert, 4 = Rather alert, 5 = Neither alert nor sleepy, 6 = Some signs of sleepiness, 7 = Sleepy, but no effort to keep awake, 8 = Sleepy, some effort to keep awake, 9 = Very sleepy, great effort to keep awake, fighting sleep. The KSS has been validated against electroencephalography (EEG) and behavioural measures of sleepiness [59]. A score of 6 and above on the KSS has been linked to significant increases in reaction time and lapses on a psychomotor vigilance task, and decreased levels of arousal as measured by the alpha attenuation test [59].

To screen for dementia and MCI, and to detect change in cognitive function associated with dementia and MCI (attention, orientation, memory, fluency, language and visuospatial abilities), the ACE-R will be administered during screening visits and at the end of each dietary phase [60]. The ACE-R is frequently used in clinical and research settings, with high sensitivity and specificity for detecting both dementia and MCI.

Future risk of dementia will be calculated using the Cardiovascular Risk Factors, Aging and Dementia (CAIDE) score, and the Framingham vascular risk score (FRS) [61]. The CAIDE predicts 20-year dementia risk based on middle age profiles with high sensitivity (0.81) and moderate specificity (0.61). The CAIDE score is based on age, sex, education, total cholesterol, systolic blood pressure, physical activity and APOEε4 genotype. Similarly, the FRS determines risk of CVD on the basis of age, sex, systolic blood pressure, hypertension, HDL cholesterol, total cholesterol, smoking status and diabetes [62]. Although originally developed to predict risk of vascular disease, the FRS reliably predicts decline in cognitive function and has demonstrated stronger associations than the CAIDE with 10-year risk of cognitive decline [10].

Psychological well-being, an outcome and determinant of health behaviours, will be evaluated through the SF-36 Health Survey Version II adapted for use in Australia [63] and the Profile of Mood States (POMS). The SF-36 and POMS have been chosen for their relevance to a non-clinical population. The SF-36 was designed to measure subjective perceptions of health status across the domains of vitality, physical functioning, bodily pain, general health perceptions, physical role functioning, emotional role functioning, social role functioning and mental health [64]. The Australian SF-36 has demonstrated high internal consistency and reliability, adequate test-retest reliability and sensitivity to change, including sensitivity to nutritional interventions [65, 66]. The POMS measures mood states of tension, anxiety, anger, hostility, vigour, activity, fatigue, inertia, depression, dejection, confusion and bewilderment by asking participants to rate the extent to which they have recently felt different emotions [67]. For the purpose of the proposed study, participants will be asked about the extent to which they have felt emotions over the past month. The POMS has demonstrated good internal consistency (Cronbach’s alpha = 0.63 to 0.96) and test re-test reliability of *r* = 0.61 to *r* = 0.69 at 6 weeks [68].

#### Dietary adherence

A 15-item Mediterranean diet adherence survey and a 9-item low-fat diet adherence survey (Appendix 6) will be administered to participants bi-weekly to capture generalised patterns of food consumption during each of the intervention phases. Each survey has been adapted from the PREDIMED study to reflect an Australian food supply and national guidelines for alcohol consumption. While on the Mediterranean diet, participants will also complete a semi-quantitative weekly checklist (Appendix 3, described above). Adherence to the dietary phase will be determined at each bi-weekly visit, where participants must meet a minimum adherence requirement of 75%, as determined by checklists. If participants scoring below 75% are unable to increase their adherence in the next 2 weeks they will be excluded from the study.

To determine the impact of dietary adherence on outcomes an overall Mediterranean diet score [15, 26] will be calculated using data from a 3-day weighed food record (WFR) completed by participants before and at the end of each intervention phase. Further, erythrocyte fatty acids will provide a biological measure of dietary adherence at the end of each intervention phase.

#### Exit survey

At the end of each intervention an exit survey will be administered to capture further insight and qualitative information about participant perceptions of the diets. The surveys will ask participants to elaborate what they enjoyed and found challenging about the diet they have just completed. To measure hunger and satiety experienced during each intervention the survey will also include five questions based on the 5-Factor Satiety Questionnaire [69] with corresponding visual analogue scales (VAS). Participants will be asked to indicate 1) “How hungry did you feel after you finished a meal?”, 2) “How full did you feel after you finished a meal”, 3) “How satisfied did you feel after you finished a meal?”, 4) “How strong was your desire to eat after you finished a meal?” and 5) “How much more could you have eaten after you finished a meal?”. For questions 1-4, the VAS will range from “Not at all” to “Extremely”, and for question 5 the VAS will range from “Nothing at all” to “A very large amount”.

### Procedure

**Fig. 2:**
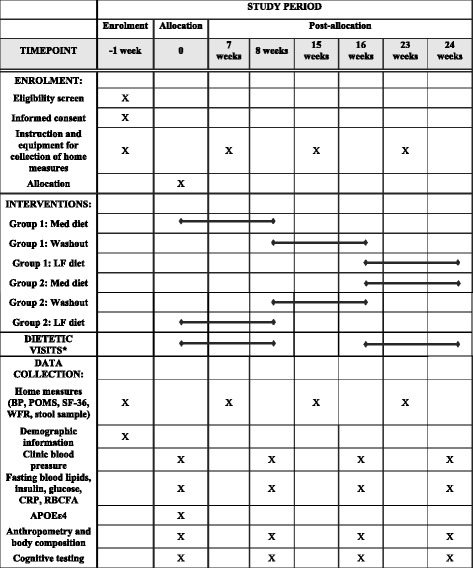
Title: Schedule of enrolment, interventions, and assessment. Legend: *Bi-weekly. Med diet, Mediterranean diet; LF, low-fat diet; BP, blood pressure; POMS, Profile of Mood States; SF-36, Short Form-36 Health Survey; WFR, weighed food record; CRP, C-reactive protein; RBCFA, red blood cell fatty acids

Data will be collected by participants at home and by study personnel during clinic assessment visits at the SIHR CTF across the course of the trial (See Fig. 2).

Participants will attend a pre-baseline appointment at the SIHR CTF where the study will be explained and informed consent will be obtained by study personnel (Appendix 7). Instructions and equipment will be given for the collection of home measures, including blood pressure, SF-36, POMS and WFR. Participants will then return 1 week later for week 0 assessments.

Clinic assessment visits will take place at baseline of the first diet (week 0), at the end of the first diet (week 8), at baseline of the second diet, after the 8-week washout period (week 16), and at the end of the second diet (week 24). Data for home measures will be collected by participants in the week prior to each of these visits.

At clinic assessment visits participants will be fasted from food, beverages (excluding water), alcohol and caffeine for 12 h. Clinic assessment visits will include measurement of blood pressure, body anthropometry, DEXA and collection of fasted blood samples. At weeks 0 and 16 participants will be allocated to their diet and meet with a dietitian to discuss the dietary guidelines. Participants will then be given a continental breakfast before cognitive tasks are administered. To minimise the impact and influence of external variables, cognitive testing conditions will be rigorously controlled and standardised as outlined by [70]. All cognitive testing sessions will take place between 9:30 am and 12:00 pm. To limit the impact of food intake on cognitive performance, participants will consume the same breakfast before each cognitive session.

If participants have performed a strenuous level of non-habitual exercise in past 10 h, not had an adequate amount of sleep, or are unusually tired cognitive assessments will be rescheduled. Temperature and noise of the cognitive testing environment will be controlled in order to reduce distraction and test administration will be standardised through the use of a testing script provided by Cambridge Cognition. Participants will commence the dietary intervention on the day following week 0 and week 16 visits, and will commence the washout period on the day of the week 8 visit.

Between weeks 0 and 8, and 16 and 24 participants will attend bi-weekly dietetic visits at the SIHR CTF. In the final dietetic visit of each diet phase, and at week 15, participants will be provided with equipment for the collection of home measures to be returned at their next clinic assessment visit.

Volunteers will be instructed to continue any habitual exercise and medications for the duration of the trial. Changes to exercise and medications will be monitored and recorded at bi-weekly dietetic visits and clinic assessment visits by the investigators.

### Statistical analysis

Statistical analyses will be conducted using SPSS for Windows, version 21.0 (SPSS Inc., Chicago, IL, USA) and Stata (version 14.2, StataCorp, College Station, TX, USA). Data will be presented as means ± standard deviation (SD) for descriptive statistics and as means ± standard error (SEM) for reporting estimated effects. All tests will be 2-tailed with *p*-values <0.05 deemed statistically significant.

Based on power calculations, a sample size of 31 volunteers is required to detect a clinically relevant difference of 2.5 mmHg in home blood pressure, the primary outcome measure, with power of 90%. This calculation accounts for a total of 54 readings (three readings taken three times per day for 6 days) at each time point, a within-group standard deviation of 14 mmHg, a within-subject repeated measures correlation of *r* = 0.6 and a between-phase within-subject correlation of *ρ* = 0.5. The correlation (ρ) and use of a cross-over design reduces the number of required participants by a factor of (1 − ρ)/2 = 4 [153] i.e., from approximately *n* = 124 for a parallel group design using ANCOVA (*n* = 62 per group) to *n* = 31 subjects in total.

Baseline demographic, cardiometabolic, cognitive, well-being and dietary characteristic data will be compared for those who complete the study and those who withdraw using independent t-test and chi-square tests, depending on the type of variable and distribution of data.

The primary analysis will be performed on a per protocol basis including only those subjects who complete both trial phases. A linear mixed effects model will determine the difference in each outcome between dietary interventions and will measure both between and within subject effects for cardiometabolic and cognitive outcomes. If a significant change is detected for cognitive outcomes a hierarchical regression will include cardiometabolic outcomes to assess their meditating effects. A sensitivity analysis will include weight loss, change in physical activity, medications and supplements as these may cause time-varying confounding should they differ between groups in the second dietary phase. Further, carryover and treatment-period effects will be assessed by including treatment*phase and treatment*order interaction terms. A diet*period*energy intake interaction term will be included to determine any significant changes between diets in relation to total energy intake, macronutrient intake and micronutrient intake. As an additional sensitivity analysis we will also include subjects that did not complete the trial or were not included in the per protocol analysis due to inadequate adherence (<75%). For missing data the linear mixed effects models will estimate unbiased effects as opposed to multiple imputation.

### Trial and data management

To ensure privacy and confidentiality each participant will be assigned a unique study identification (ID) number. All source documents will be de-identified (re-identifiable by coded ID numbers) and stored in an individual file for each study participant. All information collected as part of the study will remain confidential and no information that could lead to identification of any individual will be released. Access to trial data will be limited to the team of investigators named as authors on this publication.

Unintended effects and adverse events of the trial intervention will be identified during bi-weekly dietetic visits. All adverse and serious adverse events will be recorded and serious adverse events will be reported by the Chief Investigators to the University of South Australia Human Research Ethics Committee. Participants will be advised to consult with their General Practitioner regarding any notable symptoms identified throughout the trial. If the instance of adverse events a decision to continue, modify or cease the intervention will be made on a case-by-case basis.

Due to the size of the trial and nature of the trial intervention a Data Monitoring Committee is not deemed necessary. The Chief Investigator will conduct quality control checks of data values twice during each dietary intervention phase to ensure data quality.

Electronic data entry will be completed throughout the trial by study personnel. Electronic data files will be stored on a secure password protected network and backed up regularly on an external hard drive. Once the trial is complete and results have been published electronic files will be stored on a DVD in the Sansom Clinical Trials facility secure data store in the Bonython Jubilee Building, City East Campus. Study documentation will be kept in locked filing cabinets within in the Sansom Clinical Trials facility in the Bonython Jubilee Building, City East Campus. Once the study is complete and results have been published all data will be archived and stored for a total of 15 years.

Any changes to the trial protocol will require approval by all authors and the University of South Australia Human Ethics Committee. Once approved, modifications to the trial protocol will be communicated to the funding sponsors and the journal wherein the protocol is published.

Results from this trial will be disseminated through peer-reviewed international journals, presentations at national and international meetings and the lead author’s doctoral dissertation. Participants will be provided with a copy of their personal results within 6 months of completing the study and a summary of the research findings will be sent to all participants once data analysis has been completed.

## Discussion

To reduce the impact of both dementia and CVD health and lifestyle interventions with the potential to mitigate multiple risk factors have been recommended above reactive, costly treatments [71, 72]. Risk factors such as hypertension, dyslipidaemia, insulin sensitivity are shared by both dementia and CVD [73–76]. Interventions targeting each of these simultaneously may be effective in reducing the prevalence of both CVD and dementia. However, it is necessary to ensure that such interventions are sustainable, both for the target population and the environment.

The Mediterranean diet has demonstrated efficacy for reducing risk of CVD and cognitive decline. Yet an Australian population, who typically consumes a high quantity of meat, may find adhering to the meat restrictions of the diet difficult. This paper outlines the protocol of a randomised controlled trial that endeavours to compare a Mediterranean diet with an additional source of meat protein, against a low-fat control diet. Findings of the forthcoming study will be relevant at a policy, clinical and individual level and will provide insight as to whether consuming pork as a part of a Mediterranean diet can improve health outcomes while simultaneously increasing variety and sustainability for the target population.

## Figures and Tables

**Fig. 3:**
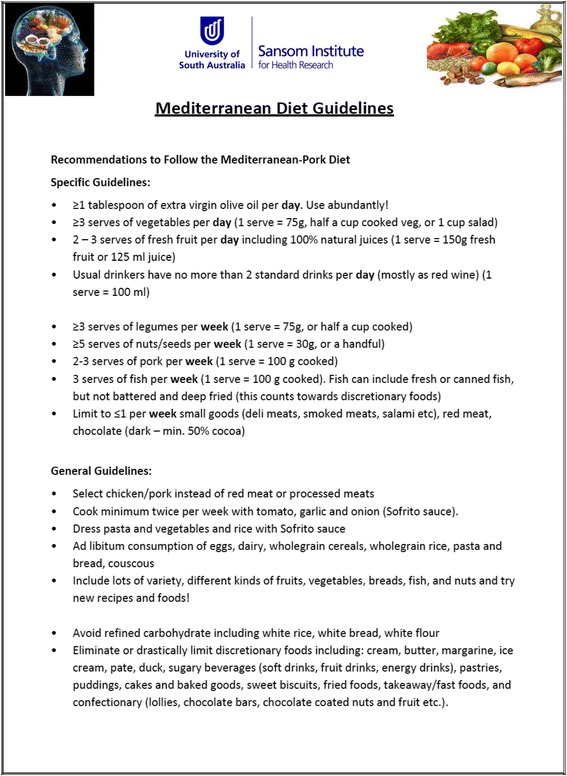
Guidelines for the MedPork intervention

**Fig. 4:**
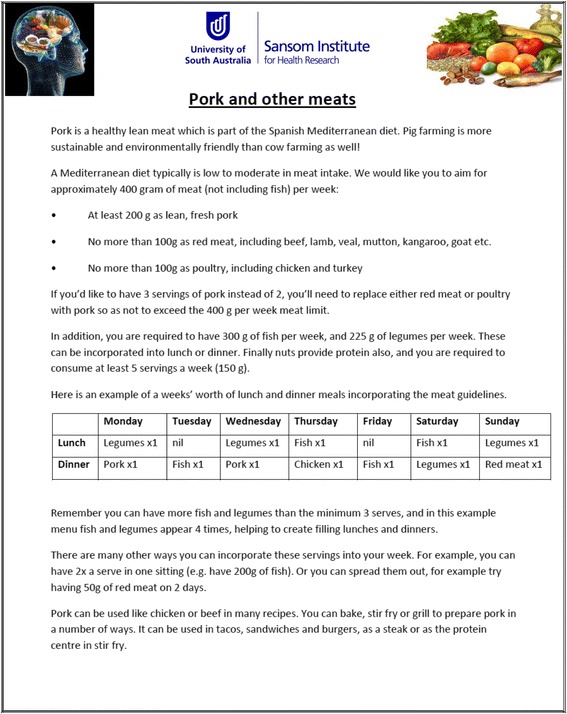
Recommendations for including pork in the Mediterranean diet

**Fig. 5:**
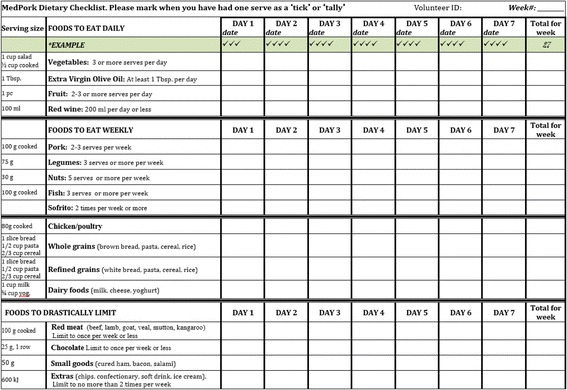
Semi-quantitative weekly checklist to encourage adherence to the MedPork intervention

**Fig. 6:**
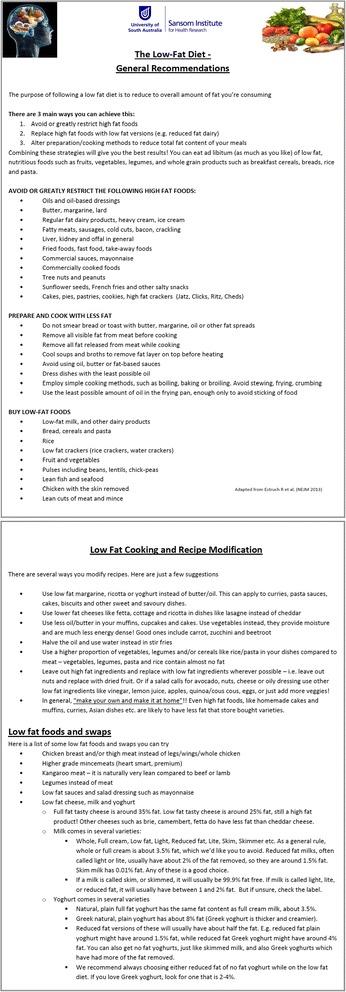
Guidelines for the LF intervention

**Fig. 7:**
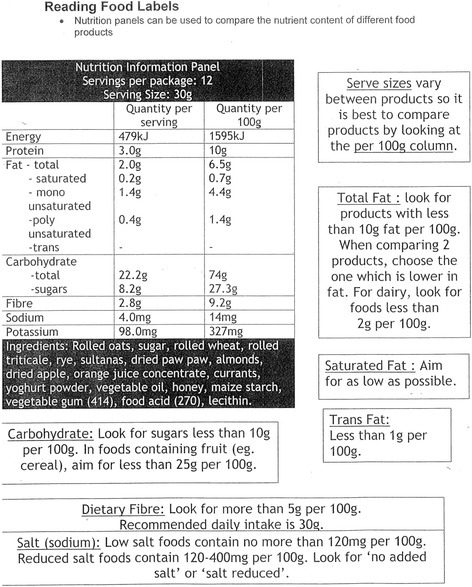
Dietetic resource for reading food labels while following the LF intervention

**Fig. 8:**
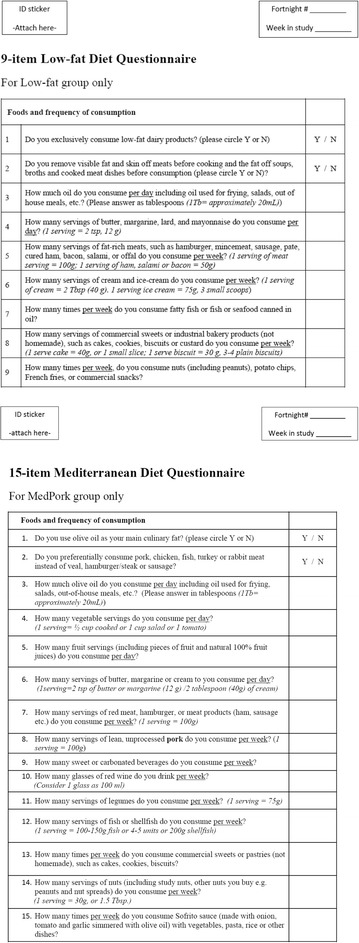
9-item Low Fat diet adherence survey and 15-item Mediterranean diet adherence survey

**Fig. 9:**
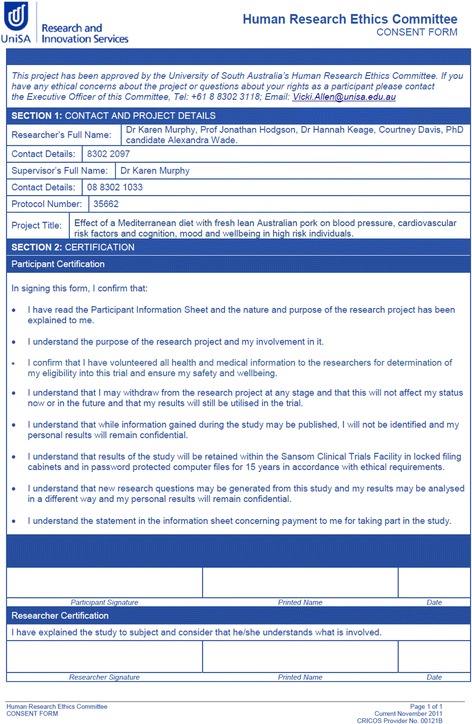
Participant consent form

## Abbreviations

ACE-R: Addenbrooke’s cognitive examination – revised
ATS: Attention switching task
BMI: Body mass index
CAIDE: Cardiovascular risk factors, aging and dementia
CANTAB: Cambridge automated neuropsychological test automated battery
CRP: C-reactive protein
CVD: Cardiovascular disease
DEXA: Dual-energy x-ray absorptiometry
DLQ: Diet and lifestyle questionnaire
DMS: Delayed matching to sample
EEG: Electroencephalography
EPIC: European prospective investigation into cancer
EVOO: Extra virgin olive oil
FRS: Framingham risk score
HDL: High-density lipoprotein
ID: Identification
KSS: Karolinska sleep scale
LDL: Low-density lipoprotein
MCI: Mild cognitive impairment
MOT: Motor orientation task
NATA: National Association of Testing Authorities, Australia
OTS: One touch stockings of cambridge
PAL: Paired associates learning
POMS: Profile of mood states
PREDIMED: Prevención con Dieta Mediterránea
RCT: Randomised controlled trial
RTI: Reaction time
RVP: Rapid visual information processing
SF-36: Short form-36 health survey
SIHR CTF: Sansom Institute for Health Research Clinical Trial Facility
SWM: Spatial working memory
T2DM: Type 2 diabetes mellitus
VAS: Visual analogue scale
VRM: Verbal recognition memory
WFR: Weighed food record

## References

[1] Prince MJ, Wu F, Guo Y, Gutierrez Robledo LM, O'Donnell M, Sullivan R, Yusuf S (2015). The burden of disease in older people and implications for health policy and practice. Lancet.

[2] Alzheimer's Disease International. World Alzheimer report 2015 the global impact of dementia. London; 2015. https://www.alz.co.uk/research/world-report-2015.

[3] Heidenreich PA, Trogdon JG, Khavjou OA, Butler J, Dracup K, Ezekowitz MD, Finkelstein EA, Hong Y, Johnston SC, Khera A (2011). Forecasting the future of cardiovascular disease in the United States: a policy statement from the American Heart Association. Circulation.

[4] World Health Organisation. Global status report on noncommunicable diseases. Geneva; 2014. http://www.who.int/nmh/publications/ncd-status-report-2014/en/.

[5] Alonso A, Jacobs DR, Menotti A, Nissinen A, Dontas A, Kafatos A, Kromhout D (2009). Cardiovascular risk factors and dementia mortality: 40 years of follow-up in the seven countries study. J Neurol Sci.

[6] Keys A, Mienotti A, Karvonen MJ, Aravanis C, Blackburn H, Buzina R, Djordjevic BS, Dontas AS, Fidanza F, Keys MH (1986). The diet and 15-year death rate in the seven countries study. Am J Epidemiol.

[7] Menotti A, Kromhout D, Blackburn H, Fidanza F, Buzina R, Nissinen A (1999). Food intake patterns and 25-year mortality from coronary heart disease: cross-cultural correlations in the seven countries study. The seven countries study research group. Eur J Epidemiol.

[8] Ng JB, Turek M, Hakim AM (2013). Heart disease as a risk factor for dementia. Clin Epidemiol.

[9] Kaffashian S, Dugravot A, Elbaz A (2013). Predicting cognitive decline: a dementia risk score vs the Framingham vascular risk scores predicting cognitive decline.

[10] Davis CR, Bryan J, Hodgson J, Murphy K (2015). Definition of the mediterranean diet: a literature review. Nutrients.

[11] Fung TT, McCullough ML, Newby PK, Manson JE, Meigs JB, Rifai N, Willett WC, Hu FB (2005). Diet-quality scores and plasma concentrations of markers of inflammation and endothelial dysfunction. Am J Clin Nutr.

[12] Hodgson JM, Croft KD, Woodman RJ, Puddey IB, Fuchs D, Draijer R, Lukoshkova E, Ga H (2013). Black tea lowers the rate of blood pressure variation : a randomized. Am J Clin Nutr.

[13] Bondonno CP, Croft KD, Ward N, Considine MJ, Hodgson JM (2015). Dietary flavonoids and nitrate: effects on nitric oxide and vascular function. Nutr Rev.

[14] Schwingshackl L, Christoph M, Hoffmann G (2015). Effects of olive oil on markers of inflammation and endothelial function: a systematic review and meta-analysis. Nutrients.

[15] Trichopoulou A, Costacou T, Bamia C, Dimitrios T (2003). Adherence to a Mediterranean diet and survival in a Greek population. N Engl J Med.

[16] Jacobs DR, Tapsell LC (2013). Food synergy: the key to a healthy diet. Proc Nutr Soc.

[17] Trichopoulou A, Vasilopoulou E, Georga K, Soukara S, Dilis V. Traditional foods : Why and how to sustain them. Trends Food Sci Technol. 2006;17:498–504. doi:10.1016/j.tifs.2006.03.005.

[18] Nissensohn M, Román-Viñas B, Sánchez-Villegas A, Piscopo S, Serra-Majem L (2016). The effect of the Mediterranean diet on hypertension: a systematic review and meta-analysis. J Nutr Educ Behav.

[19] Richard C, Couture P, Desroches S, Lamarche B (2012). Effect of the Mediterranean diet with and without weight loss on markers of inflammation in men with metabolic syndrome. Obesity.

[20] Vincent-baudry S, Defoort C, Gerber M, Bernard M-c, Verger P, Helal O (2005). The Medi-RIVAGE study : reduction of cardiovascular disease risk factors after a 3-mo intervention with a Mediterranean-type diet or a.

[21] Estruch R, Ros E, Salas-Salvadó J, Covas M-I, Corella D, Arós F, Gómez-Gracia E, Ruiz-Gutiérrez V, Fiol M, Lapetra J (2013). Primary prevention of cardiovascular disease with a Mediterranean diet. N Engl J Med.

[22] Chrysohoou C, Panagiotakos DB, Pitsavos C, Das UN, Stefanadis C (2004). Adherence to the Mediterranean diet attenuates inflammation and coagulation process in healthy adults: the ATTICA study. J Am Coll Cardiol.

[23] Sofi F, Cesari F, Abbate R, Gensini GF, Casini A (2008). Adherence to Mediterranean diet and health status: meta-analysis. BMJ (Clinical research ed).

[24] Féart C, Samieri C, Rondeau V, Amieva H, Portet F, Dartigues J-F, Scarmeas N, Barberger-Gateau P (2009). Adherence to a Mediterranean diet, cognitive decline, and risk of dementia. JAMA.

[25] Gu Y, Luchsinger JA, Stern Y, Scarmeas N (2010). Mediterranean diet, inflammatory and metabolic biomarkers, and risk of Alzheimer's disease. J Alzheimers Dis.

[26] Davis CR, Bryan J, Hodgson JM, Wilson C, Dhillon V, Murphy K (2015). A randomised controlled intervention trial evaluating the efficacy of a Mediterranean dietary pattern on cognitive function and psychological wellbeing in healthy older adults: the MedLey study. BMC Geriatr.

[27] Davis CR, Hodgson J, Bryan J, Woodman RJ, Murphy K (2017). Older Australians can achieve high adherence to the Mediterranean diet during a 6 month intervention; results from the Medley study.

[28] Ministry of Health and Welfare SSHC (1999). Dietary guidelines for adults in Greece. Arch Hell Med.

[29] Bach-Faig A, Berry EM, Lairon D, Reguant J, Trichopoulou A, Dernini S, Medina FX, Battino M, Belahsen R, Miranda G (2011). Mediterranean diet pyramid today. Science and cultural updates. Public Health Nutr.

[30] Australian Bureau of Statistics (2016). Australian health survey: consumption of food groups from the Australian dietary guidelines.

[31] Organisation for Economic C-o, Development (2016). Agricultural output - Meat consumption.

[32] Knight A, Bryan J, Murphy K (2016). Is the Mediterranean diet a feasible approach to preserving cognitive function and reducing risk of dementia for older adults in western countries? New insights and future directions. Ageing Res Rev.

[33] Wolk A (2017). Potential health hazards of eating red meat. J Intern Med.

[34] Van Hecke T, Van Camp J, De Smet S (2017). Oxidation during digestion of meat: interactions with the diet and helicobacter pylori gastritis, and implications on human health. Compr Rev Food Sci Food Saf.

[35] Tirosh O, Shpaizer A, Kanner J (2015). Lipid peroxidation in a stomach medium is affected by dietary oils (olive/fish) and antioxidants: the Mediterranean versus western diet. J Agric Food Chem.

[36] Norat T, Bingham S, Ferrari P, Slimani N, Jenab M, Mazuir M, Overvad K, Olsen A, Tjonneland A, Clavel F (2005). Meat, fish, and colorectal cancer risk: the European prospective investigation into cancer and nutrition. J Natl Cancer Inst.

[37] Cross AJ, Harnly JM, Ferrucci LM, Risch A, Mayne ST, Sinha R (2012). Developing a heme iron database for meats according to meat type, cooking method and doneness level. Food Nutr Sci.

[38] Murphy KJ, Thomson RL, Coates AM, Buckley JD, Howe PRC (2012). Effects of eating fresh lean pork on cardiometabolic health parameters. Nutrients.

[39] Tilman D, Clark M (2014). Global diets link environmental sustainability and human health. Nature.

[40] National Vascular Disease Prevention Alliance (2012). Guidelines for the management of absolute cardiovascular disease risk.

[41] Desideri G, Kwik-Uribe C, Grassi D, Necozione S, Ghiadoni L, Mastroiacovo D, Raffaele A, Ferri L, Bocale R, Lechiara MC (2012). Benefits in cognitive function, blood pressure, and insulin resistance through cocoa flavanol consumption in elderly subjects with mild cognitive impairment: the cocoa, cognition, and aging (CoCoA) study. Hypertension.

[42] Mastroiacovo D, Kwik-Uribe C, Grassi D, Necozione S, Raffaele A, Pistacchio L, Righetti R, Bocale R, Lechiara MC, Marini C (2015). Cocoa flavanol consumption improves cognitive function, blood pressure control, and metabolic profile in elderly subjects: the cocoa, cognition, and aging (CoCoA) study—a randomized controlled trial 1–4. Am J Clin Nutr.

[43] Nilsson A, Radeborg K, Salo I, Björck I (2012). Effects of supplementation with n-3 polyunsaturated fatty acids on cognitive performance and cardiometabolic risk markers in healthy 51 to 72 years old subjects: a randomized controlled cross-over study. Nutr J.

[44] Cao J, Schwichtenberg KA, Hanson NQ, Tsai MY (2006). Incorporation and clearance of omega-3 fatty acids in erythrocyte membranes and plasma phospholipids. Clin Chem.

[45] Martínez-González MA, Corella D, Salas-salvadó J, Ros E, Covas MI, Fiol M, Wärnberg J, Arós F, Ruíz-Gutiêrrez V, Lamuela-Raventós RM (2012). Cohort profile: design and methods of the PREDIMED study. Int J Epidemiol.

[46] The Australian National Health and Medical Research Council. Australian guidelines to reduce health risks from drinking alcohol. Canberra, ACT: The Australian National Health and Medical Research Council; 2009.

[47] Ohkubo T, Imai Y, Tsuji I, Nagai K, Kato J, Kikuchi N, Nishiyama A, Aihara A, Sekino M, Kikuya M (1998). Home blood pressure measurement has a stronger predictive power for mortality than does screening blood pressure measurement: a population-based observation in Ohasama. Jpn J Hypertens.

[48] Jula A, Puukka P, Karanko H (1999). Multiple clinic and home blood pressure measurements versus ambulatory blood pressure monitoring. Hypertension.

[49] Mule G, Caimi G, Cottone S, Nardi E, Andronico G, Piazza G, Volpe V, Federico MR, Cerasola G (2002). Value of home blood pressures as predictor of target organ damage in mild arterial hypertension. J Cardiovasc Risk.

[50] Koch W, Ehrenhaft A, Griesser K, Pfeufer A, Müller J, Schömig A, Kastrati A. TaqMan Systems for Genotyping of disease-related polymorphisms present in the gene encoding Apolipoprotein E. Clin Chem Lab Med. 2002;40(11):1123–31. 10.1515/CCLM.2002.197 12521230

[51] Pase MP, Grima N, Cockerell R, Stough C, Scholey A, Sali A, Pipingas A (2015). The effects of long-chain omega-3 fish oils and multivitamins on cognitive and cardiovascular function: a randomized, controlled clinical trial. J Am Coll Nutr.

[52] Yurko-Mauro K, Alexander DD, Van Elswyk ME (2015). Docosahexaenoic acid and adult memory: a systematic review and meta-analysis. PLoS One.

[53] Amen DG, Taylor DV, Ojala K, Kaur J, Willeumier K (2013). Effects of brain-directed nutrients on cerebral blood flow and neuropsychological testing: a randomized, double-blind, placebo-controlled, crossover trial. Adv Mind Body Med.

[54] Bauer I, Hughes M, Rowsell R, Cockerell R, Pipingas A, Crewther S, Crewther D (2014). Omega-3 supplementation improves cognition and modifies brain activation in young adults. Hum Psychopharmacol Clin Exp.

[55] File SE, Jarrett N, Fluck E, Duffy R, Casey K, Wiseman H (2001). Eating soya improves human memory. Psychopharmacology.

[56] Strike SC, Carlisle A, Gibson EL, Dyall SC (2016). A high omega-3 fatty acid multinutrient supplement benefits cognition and mobility in older women: a randomized, double-blind, placebo-controlled pilot study. J Gerontol Ser A Biol Med Sci.

[57] Louis WJ, Mander AG. Use of computerized neuropsychological tests (CANTAB) to assess cognitive effects of antihypertensive drugs in the elderly. J hypertens. 1999;17(12 part 2):1813–9. 10.1097/00004872-199917121-00005 10703873

[58] Cambridge Cognition (2008). Test-retest reliabilities and detecting reliable change.

[59] Kaida K, Takahashi M, Åkerstedt T, Nakata A, Otsuka Y, Haratani T, Fukasawa K (2006). Validation of the Karolinska sleepiness scale against performance and EEG variables. Clin Neurophysiol.

[60] Mioshi E, Dawson K, Mitchell J, Arnold R, Hodges JR (2006). The Addenbrooke’s cognitive examination revised (ACE-R): a brief cognitive test battery for dementia screening. Int J Geriatr Psychiatry.

[61] Kivipelto M, Ngandu T, Laatikainen T, Winblad B, Soininen H, Tuomilehto J (2006). Risk score for the prediction of dementia risk in 20 years among middle aged people: a longitudinal, population-based study. Lancet Neurol.

[62] D'Agostino RB, Vasan RS, Pencina MJ, Wolf PA, Cobain M, Massaro JM, Kannel WB (2008). General cardiovascular risk profile for use in primary care: the Framingham heart study. Circulation.

[63] Sanson-Fisher RW, Perkins JJ (1998). Adaptation and validation of the SF-36 health survey for use in Australia. J Clin Epidemiol.

[64] Ware JE (1994). SF-36 physical and mental health summary scales: a user's manual.

[65] Crichton GE, Bryan J, Murphy KJ (2013). Dietary antioxidants, cognitive function and dementia - a systematic review. Plant Foods Hum Nutr.

[66] Sinn N, Milte CM, Street SJ, Buckley JD, Coates AM, Petkov J, Howe PRC (2012). Effects of n-3 fatty acids, EPA v. DHA, on depressive symptoms, quality of life, memory and executive function in older adults with mild cognitive impairment: a 6-month randomised controlled trial. Br J Nutr.

[67] McNair DM, Lorr M, Droppleman LF (1971). Manual for the profile of mood states san Diego CA: educational and industrial testing services.

[68] Curran SL, Andrykowski MA, Studts JL (1995). Short form of the profile of mood states (POMS-SF): psychometric information. Psychol Assess.

[69] Karalus M, Vickers Z (2016). Satiation and satiety sensations produced by eating oatmeal vs. oranges. A comparison of different scales. Appetite.

[70] Stonehouse W, Ca C, Podd J, Hill SR, Minihane AM, Haskell C, Kennedy D (2013). DHA supplementation improved both memory and reaction time in healthy young adults : a randomized controlled trial 1 – 3. Am J Clin Nutr.

[71] Mendis S, Puska P, Norrving B. Global atlas on cardiovascular disease prevention and control. Geneva; 2011. p. 2–14 http://apps.who.int/mwg-internal/de5fs23hu73ds/progress?id=pmwdrK_r0pA1U5hfyio65VwgijHsa0Eo2CKlpPAl_yw.

[72] Norton S, Matthews FE, Barnes DE, Yaffe K, Brayne C (2014). Potential for primary prevention of Alzheimer's disease: an analysis of population-based data. Lancet Neurol.

[73] Libby P, Ridker PM, Maseri A (2002). Inflammation and atherosclerosis. Circulation.

[74] Hofman A, Ott A, Breteler MM, Bots ML, Slooter aJ, van Harskamp F, van Duijn CN, Van Broeckhoven C, Grobbee DE (1997). Atherosclerosis, apolipoprotein E, and prevalence of dementia and Alzheimer's disease in the Rotterdam study. Lancet.

[75] Debette S, Seshadri S, Beiser A, Au R, Himali JJ, Palumbo C, Wolf PA, DeCarli C (2011). Midlife vascular risk factor exposure accelerates structural brain aging and cognitive decline. Neurology.

[76] Raz N, Rodrigue KM, Acker JD (2003). Hypertension and the brain: vulnerability of the prefrontal regions and executive functions. Behav Neurosci.

